# Positive and negative personality descriptors: UK dataset of self-referential valence, imageability and subjective frequency ratings of 300 adjectives for use in cognitive-emotional tasks

**DOI:** 10.1016/j.dib.2022.108831

**Published:** 2022-12-16

**Authors:** Andreea Raslescu, Sophie Kreicker, Amy L Gillespie, William Berners-Lee, Susannah E Murphy, Catherine J Harmer

**Affiliations:** Department of Psychiatry, University of Oxford

**Keywords:** Emotional cognition, Cognitive tasks, Self-referential processing, Emotional words, Emotional adjectives, Personality descriptors, Self-referential valence, Word imageability, Subjective word frequency

## Abstract

Experimental tasks comparing participants’ performance for categorising, remembering, and recognising positive and negative words are widely used in the emotional cognitive domain. Such tasks are commonly used in experimental psychology and psychiatry research, and have been shown to be sensitive biomarkers of depression and antidepressant drug action [1,2]. In addition, several of these tasks investigate self-referential processing i.e., the processing of information relevant to oneself; this has been shown to modify the way emotional words are encoded and remembered and may be a target that is amenable to treatment [3,4].

In practice, the development of such tasks for implementation in research studies often depends on the selection and matching of words according to characteristics such as valence or arousal, imageability, word frequency and word length to investigate differences in a chosen domain of interest whilst keeping important confounds constant. This introduces a need for ratings covering a range of word attributes that have been shown to affect processing. In particular, ratings of self-referential valence (how positively or negatively subjects feel about a word when this is used to describe themselves/their circumstances) have been seldom included in databases, despite the frequent investigation of the concept in research [1,5]. Other important attributes often considered in the process of matching and selection are word imageability and subjective frequency [6,7].

To facilitate the word selection and matching process required in cognitive-emotional task development, the present dataset provides subjective ratings for 150 positive and 150 negative adjectives describing personality characteristics. Across four online surveys, the 300 words were rated on self-referential valence, imageability and subjective frequency by representative samples of 200 UK-based, English-speaking adults. Basic demographics and data on depressive symptoms and state anxiety were collected from all participants.

Comprehensive descriptive statistics and word length were calculated for each of the 300 words. All data cleaning and statistical analysis was performed in R.

Our work is based on years of experience using the Oxford Emotional Task Battery [1,5] and may be particularly relevant for researchers using self-referential cognitive tasks with UK-based samples.

**Specifications Table**

**Table:** 

Subject	Experimental and Cognitive Psychology
Specific subject area	Cognitive neuroscience, experimental psychology, emotional cognition
Type of data	Tables Figures
How data were acquired	Online surveys delivered via Qualtrics
Data format	Raw Analysed
Parameters for data collection	Participants were recruited via Prolific Academic. Conditions for survey completion included being over 18 years of age, being a current UK resident, being fluent in English and using a desktop computer.
Description of data collection	An initial sample of 100 participants provided self-referential valence ratings for a list of 482 adjectives depicting personality characteristics. These ratings were averaged across the sample to facilitate the exclusion of ambiguous words rated neither negative nor positive and produce a final list of 300 words (150 negative and 150 positive). We sought to further characterise these 300 words with three separate online surveys collecting ratings of self-referential valence, imageability and subjective frequency. A further 102 participants provided self-referential valence ratings, 200 participants provided imageability ratings and 202 participants provided subjective frequency ratings. Basic demographics and data on depressive symptoms and state anxiety were collected from all participants across all four surveys. No participant completed more than one of the surveys; the samples for each of the four surveys are independent.
Data source location	Institution: University of Oxford Country: United Kingdom
Data accessibility	Data hosted in a public repository. Repository name: Mendeley Data Data identification number: DOI:10.17632/kgk3jbx9xb.1 Direct URL to data: https://data.mendeley.com/datasets/kgk3jbx9xb/1

## Value of the Data

• This dataset provides ratings of self-referential valence, word imageability and subjective frequency which can be used in the development of experimental cognitive tasks for psychological and psychiatric research.
• These data are primarily of interest to researchers working in emotional information processing, either as a standalone field, for understanding psychiatric conditions, or for assessment of drug efficacy.
• The words in this dataset can be used for the development or validation of existing or novel experimental tasks used in a wide range of cognition research.
• Our focus on self-referential valence rather than more traditional arousal ratings (i.e., where subjects rate the feeling elicited by a word) was motivated by feedback from study participants completing specific self-referential emotional cognition tasks [1,5]. We believe this to be a strength of the current dataset as self-referential processes are frequently studied in psychological and psychiatric research, but self-referential ratings are seldom included in large databases. Thus, the current dataset provides a valuable resource which may be used alongside more comprehensive norms such as the English Word Database of EMOtional Terms (EMOTE) database [8].

## 1 Data Description

Positive and negative personality descriptor words dataset: calculated self-referential valence, imageability and subjective frequency statistics (central tendency, range, and variability) and word length for final list of 300 adjectives

Positive and negative personality descriptor words data dictionary: data dictionary for “Positive and negative personality descriptor words dataset”

Raw dataset 1: initial self-referential valence ratings for list of 482 adjectives, demographics, and depression and trait anxiety questionnaire responses collected from 100 participants + data dictionary

Raw dataset 2: further self-referential valence ratings for final list of 300 adjectives, demographics, and depression and trait anxiety questionnaire responses collected from 102 participants + data dictionary

Raw dataset 3: imageability ratings for final list of 300 adjectives, demographics, and depression and trait anxiety questionnaire responses collected from 200 participants + data dictionary

Raw dataset 4: subjective frequency ratings for final list of 300 adjectives, demographics, and depression and trait anxiety questionnaire responses collected from 202 participants + data dictionary

Self-referential valence reliability dataset: results from analysis of variance exploring the effects of data collection phase on self-referential valence ratings for final list of 300 adjectives + data dictionary

## 2 Experimental Design, Materials and Methods

An initial list of adjectives denoting personality characteristics was drawn up by the research team using previous rating studies [9] and online repositories. Since our focus was on building a dataset of recognisable words that could be used in cognitive assessments with varied participant samples, words that were independently identified by multiple members of the research team as inappropriate or excessively obscure were removed. This resulted in an initial list of 482 personality characteristics.

In Phase 1 of data collection, we included these 482 adjectives in an initial Qualtrics survey (see Supplementary file 1) to collect self-referential valence ratings from a representative sample of UK-based, English-speaking adults. Survey instructions were adapted from Anderson [9] with one notable change; whilst Anderson advised participants to rate the word according to how much they would like a person described using the word, we instructed participants to think of another person describing *them* as each of the words and to rate their feeling towards being described as such. The motivation for this change was to enable directly employing the resulting dataset in self-referential cognitive tasks such as the Emotional Categorisation Task of the Oxford Emotional Test Battery [1,7], where participants are given equivalent instructions when sorting words presented on screen.

Participants were thus presented with each of the 482 adjectives and asked to rate how they would feel about being described in this way, from 0 (most negative) to 100 (most positive). If they did not know the meaning of a word, they were instructed to leave the rating at the default score of 50. The order of word presentation was randomised for each participant. Throughout the survey, participants had to answer 9 simple arithmetic questions (e.g., what is 50 + 10?) intended to check their engagement in the task. We also collected basic demographic data. Survey responders also completed self-report measures of current depressive symptomatology (Center for Epidemiologic Studies Depression (CESD) scale) and trait anxiety (Trait subscale of the State-Trait Anxiety Inventory (STAI-T)). Phase 1 data collection ran between 17-18 August 2020 and 100 responders completed the survey.

**Fig. 1:**
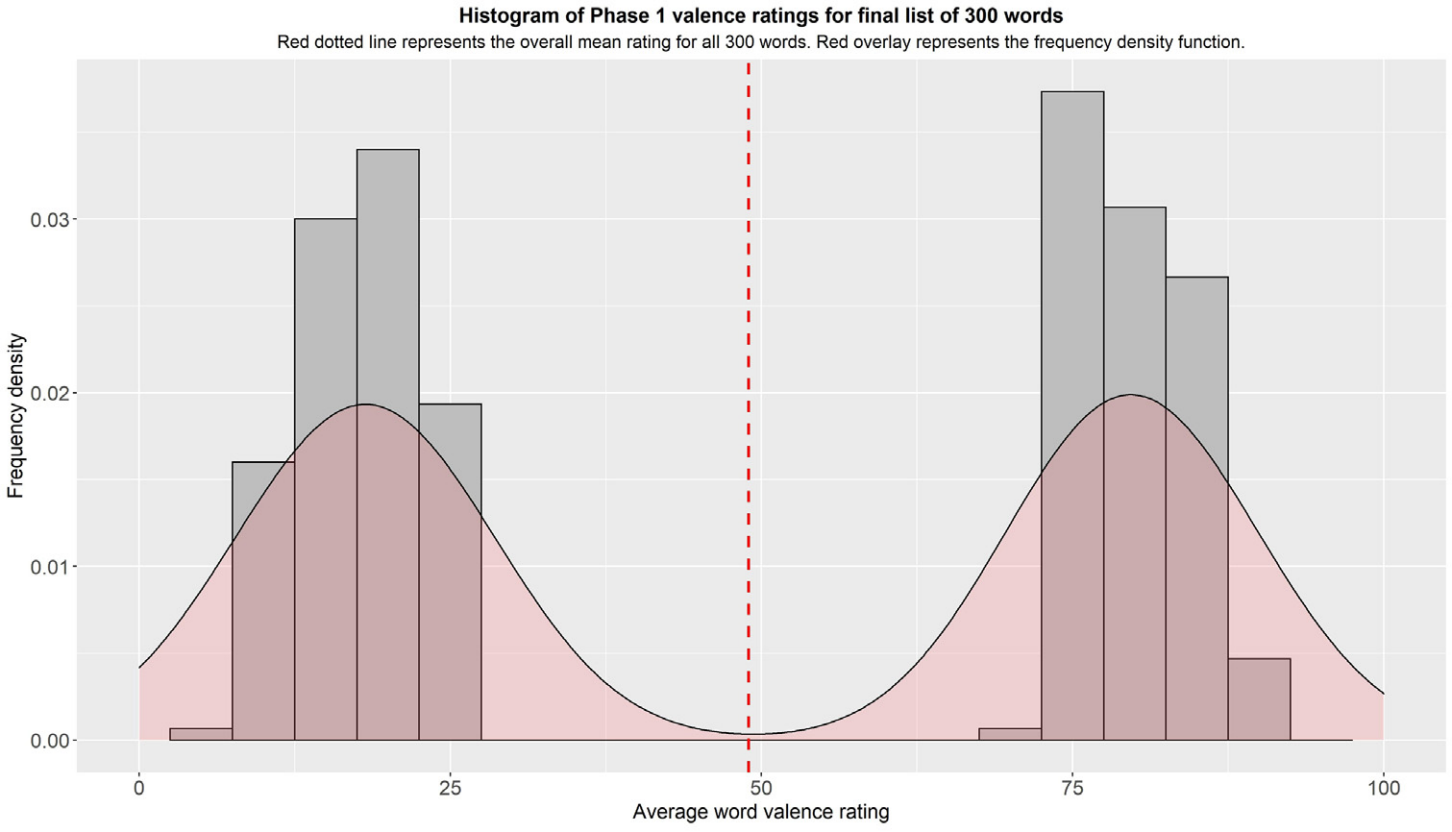
Frequency histogram of self-referential valence ratings for final list of 300 adjectives.

Data from Phase 1 was downloaded from Qualtrics (see Raw dataset 1) and processed in R (see Supplementary file 2). Survey responses were checked for completeness and proportion of engagement questions answered correctly, but none were excluded from the subsequent analysis. The maximum number of missing responses from any one participant was 83 (17%), and all engagement scores were above our cut-off of 50% correct. CESD and STAI-T responses were converted to numerical values. We computed a series of statistics (mean, standard deviation, standard error, number of ratings received, median, minimum rating, maximum rating, range, skew, kurtosis, word length) for each of the 482 adjectives and arranged them by their mean self-referential valence rating in ascending order, from most negative to most positive. We then picked the 150 most negative and 150 most positive adjectives to further characterise for our final dataset. All negative words had a self-referential valence rating of under 30, and all positive words had a self-referential valence rating of over 70. The frequency histogram of self-referential valence ratings for the final list of 300 words is displayed in Fig. 1 – the dip in the centre implies an absence of neutral words. The minimum number of ratings received for any included word was 87/100.

In Phase 2 of data collection, the resulting list of 300 words was used in three further Qualtrics surveys (see Supplementary file 1). The first sought to validate the self-referential valence ratings acquired in the initial survey and was identical in the questions it posed to participants. The second survey was used to collect word imageability ratings. Survey instructions were adapted from Cortese and Fugett [10]; responders were asked to rate how easily they could form a mental image of each of the 300 adjectives, from 0 (very difficult) to 100 (very easy). The third survey was used to collect subjective word frequency ratings. Survey instructions were adapted from Stadthagen-Gonzalez and Davis [11]; responders were asked to rate how often they came across each of the 300 adjectives in their everyday life, from 0 (never) to 100 (several times a day). For these three surveys, if they did not know the meaning of a word, participants were instructed to tick a box next to each item labelled “word not known”. These responses were stored as missing ratings and counted towards exclusions based on completeness. All three surveys featured 8 arithmetic engagement questions, demographics questions as well as the CESD and STAI-T scales. The order of word presentation was randomised for each participant. 102 responders completed the additional self-referential valence survey, 200 completed the imageability survey and 202 completed the subjective frequency survey. Phase 2 data collection ran between 24 February and 9 March 2021.

Data from Phase 2 was downloaded from Qualtrics (see Raw datasets 2-4) and separately processed in R (see Supplementary file 2). Survey responses were checked for completeness and proportion of engagement questions answered correctly. Four survey responses (three for the imageability survey and one for the subjective frequency survey) were excluded from analysis because the proportion of engagement questions answered correctly was lower than 50%. CESD and STAI-T responses were converted to numerical values. Cleaned data from the two self-referential valence surveys were merged prior to additional analyses being performed, resulting in valence ratings from 202 participants. Preliminary mean values for the self-referential valence, imageability and subjective frequency ratings of each of the 300 adjectives were calculated.

For each type of rating collected (self-referential valence, imageability, subjective frequency), we computed a “participant difference score”, as follows: for every word, we calculated the difference between each participants’ rating and the overall mean rating for that word. For every participant, we summed up those differences between their rating and the overall rating and divided the result by the number of words the participant had rated. Thus, this “participant difference score” was a measure of how much, on average, any participant's ratings deviated from the average group rating. We excluded participants whose “difference scores” were more than 3 standard deviations above the mean difference score value; this resulted in three responses being excluded from the self-referential valence survey and four responses being excluded from the subjective frequency survey.

**Fig. 2:**
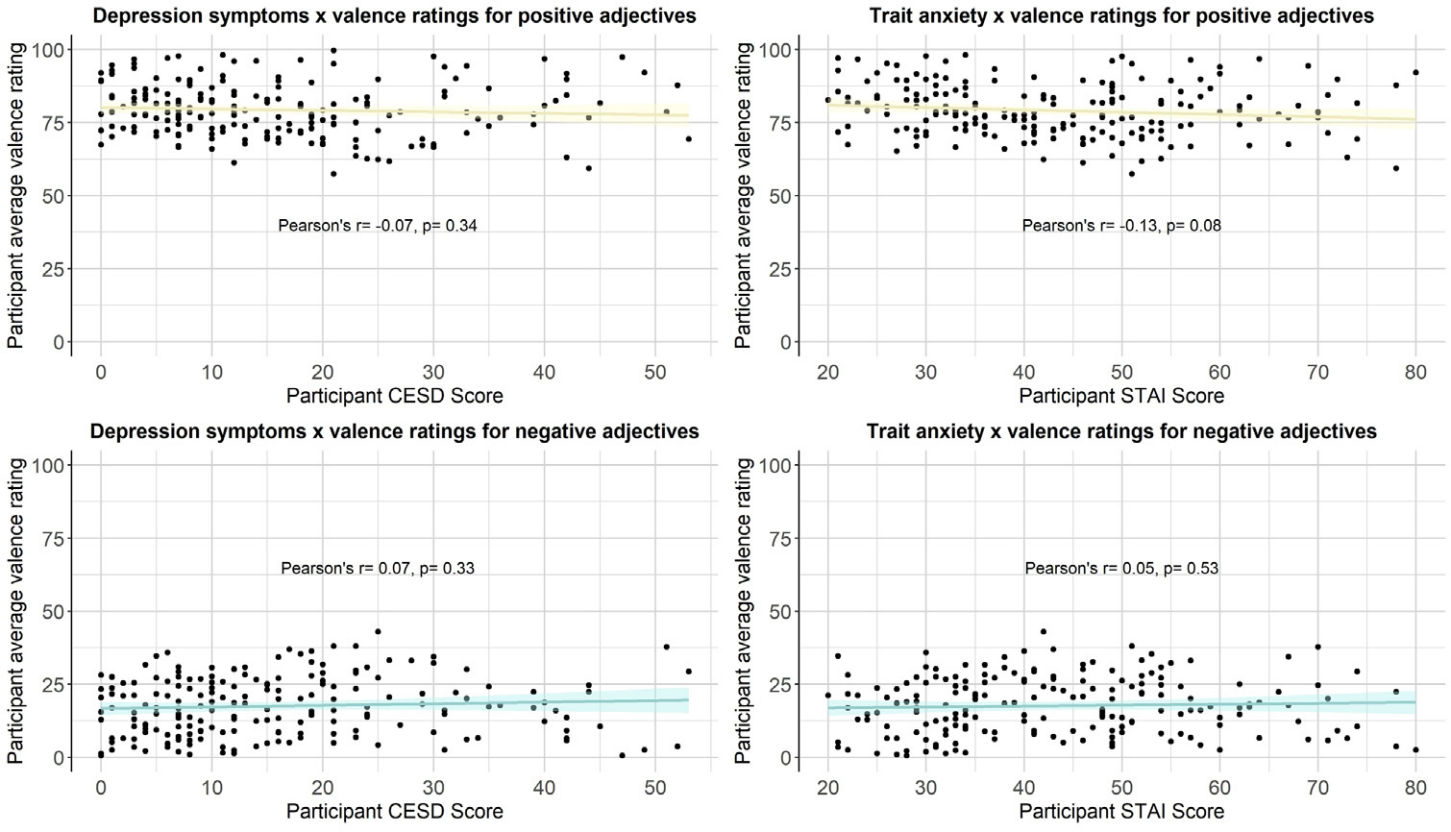
Correlations between participant self-referential valence ratings for positive/negative adjectives and their CESD/STAI-T scores.

**Fig. 3:**
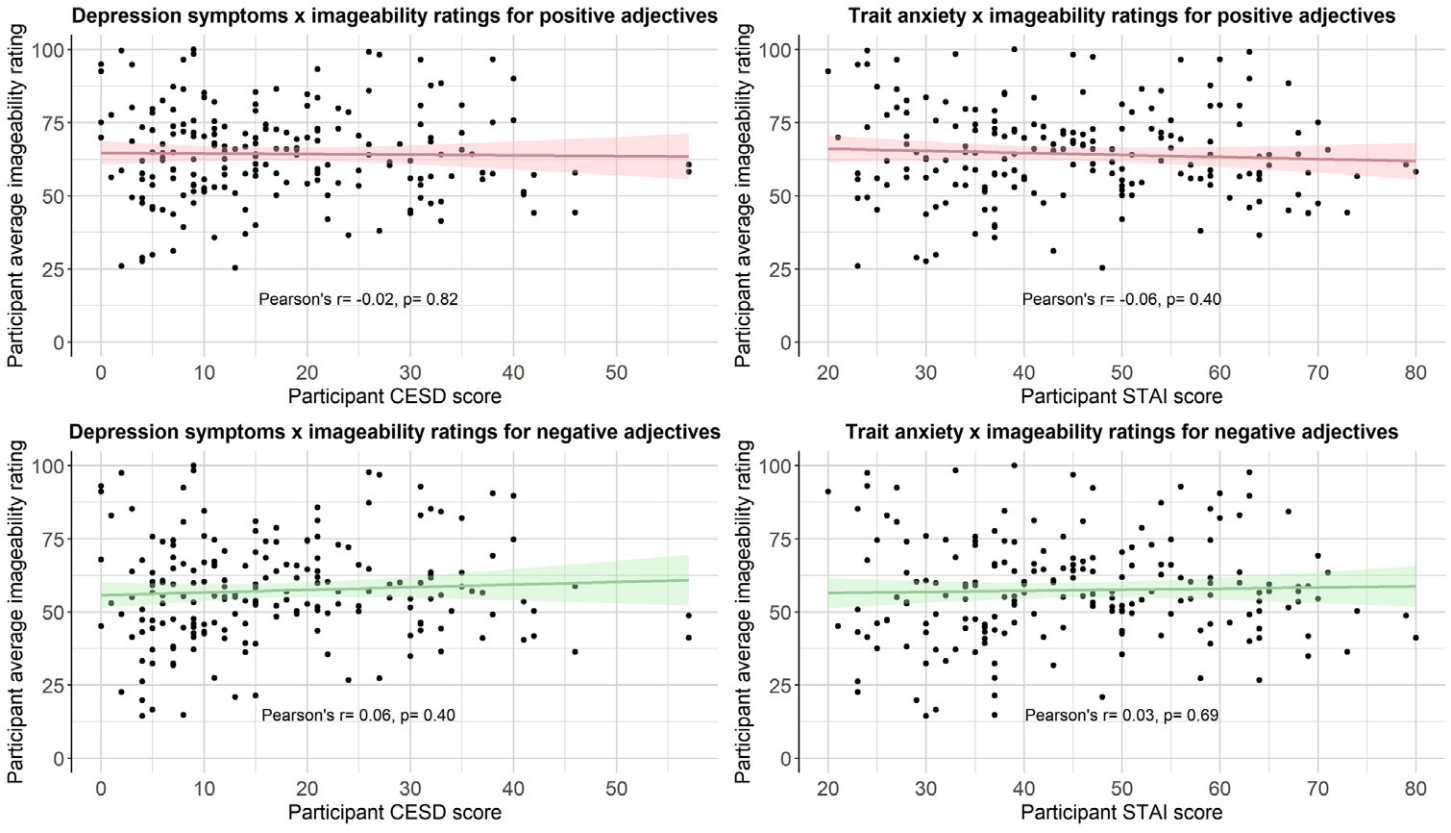
Correlations between participant imageability ratings for positive/negative adjectives and their CESD/STAI-T scores.

**Fig. 4:**
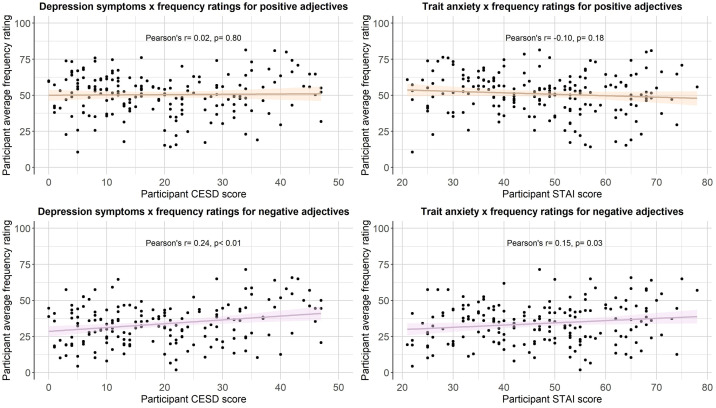
Correlations between participant subjective frequency ratings for positive/negative adjectives and their CESD/STAI-T scores.

**Fig. 5:**
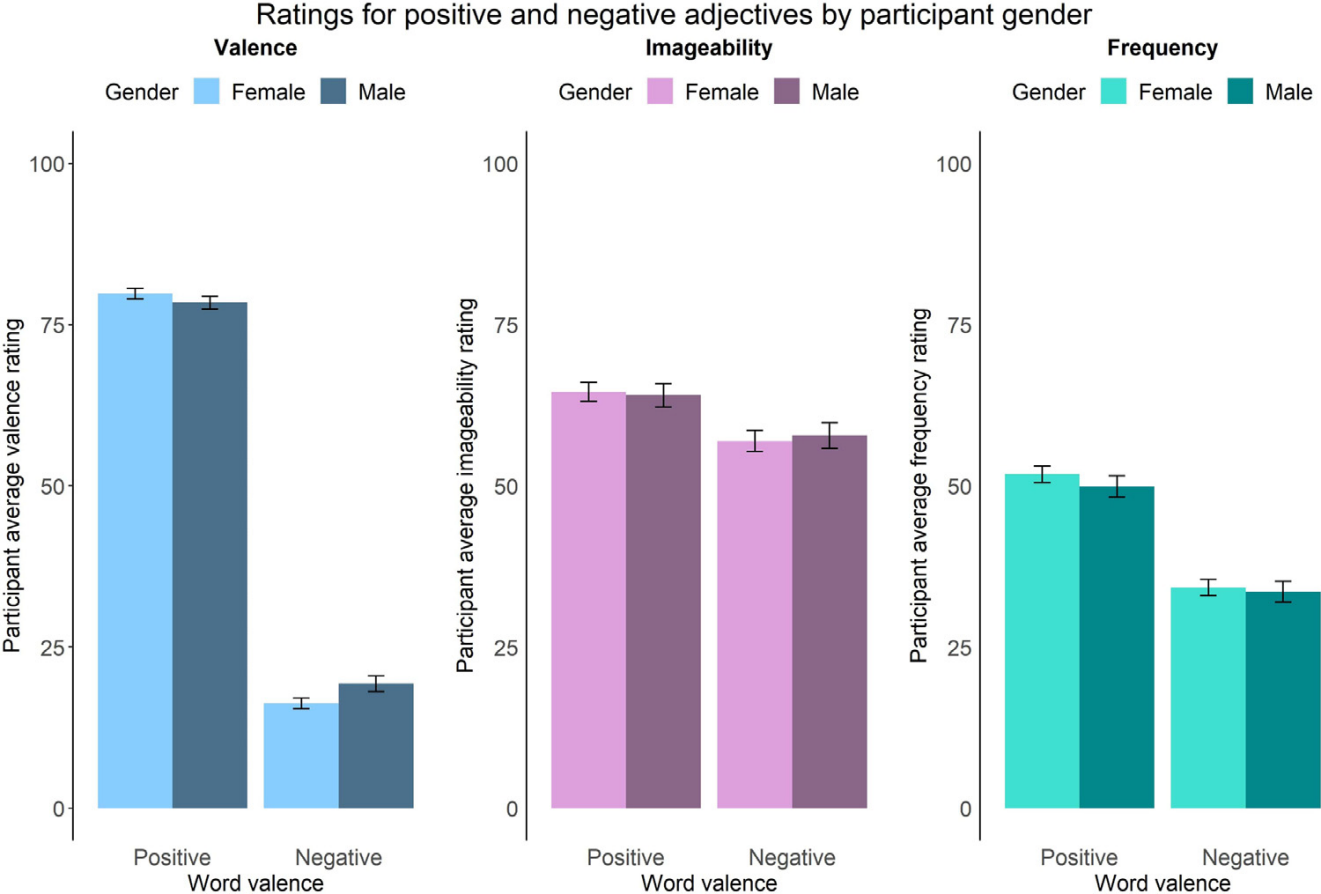
Mean self-referential valence, imageability and subjective frequency ratings for positive and negative adjectives, split by participant gender.

**Fig. 6:**
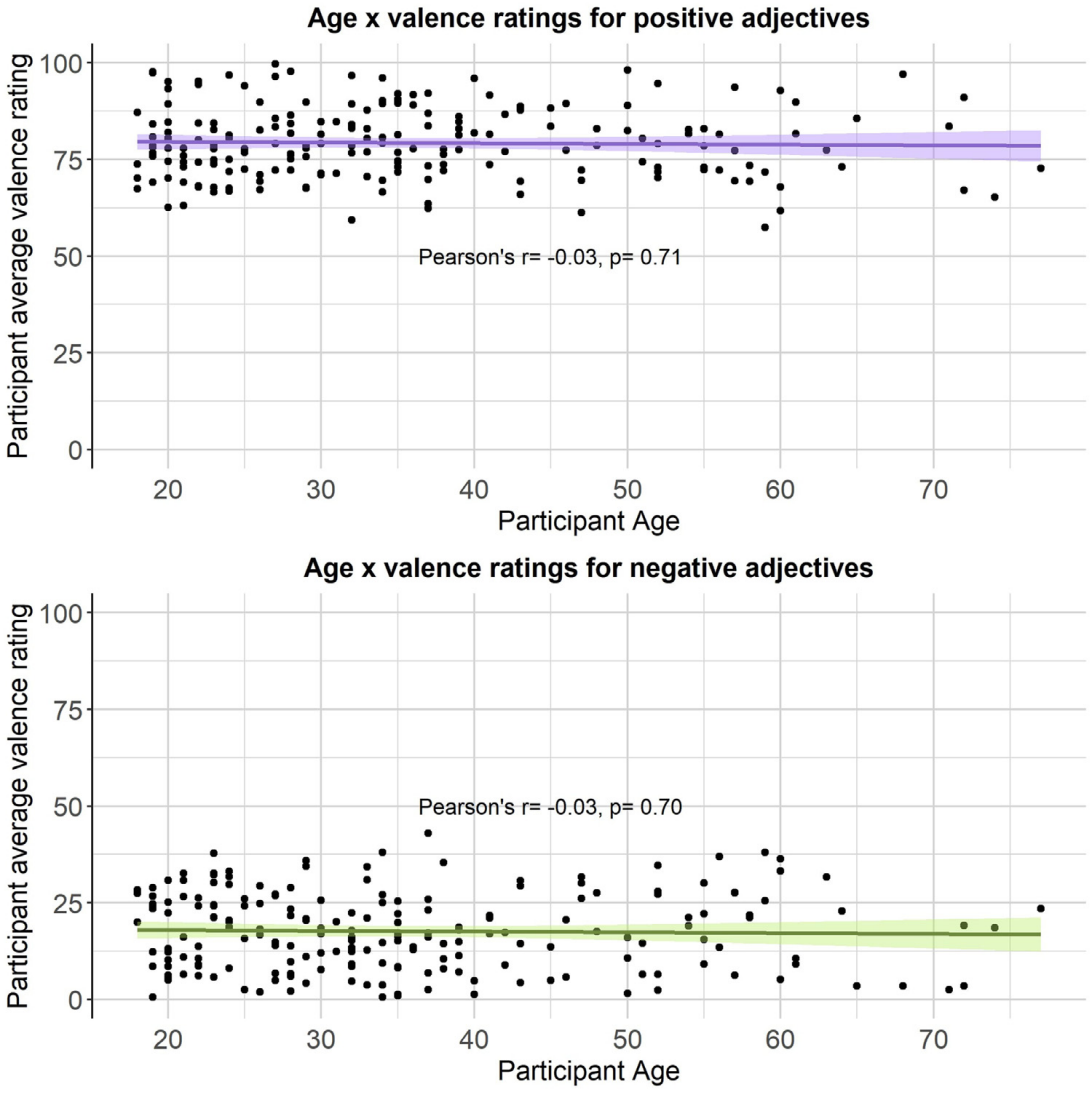
Correlations between participant self-referential valence ratings for positive/negative adjectives and their age.

**Fig. 7:**
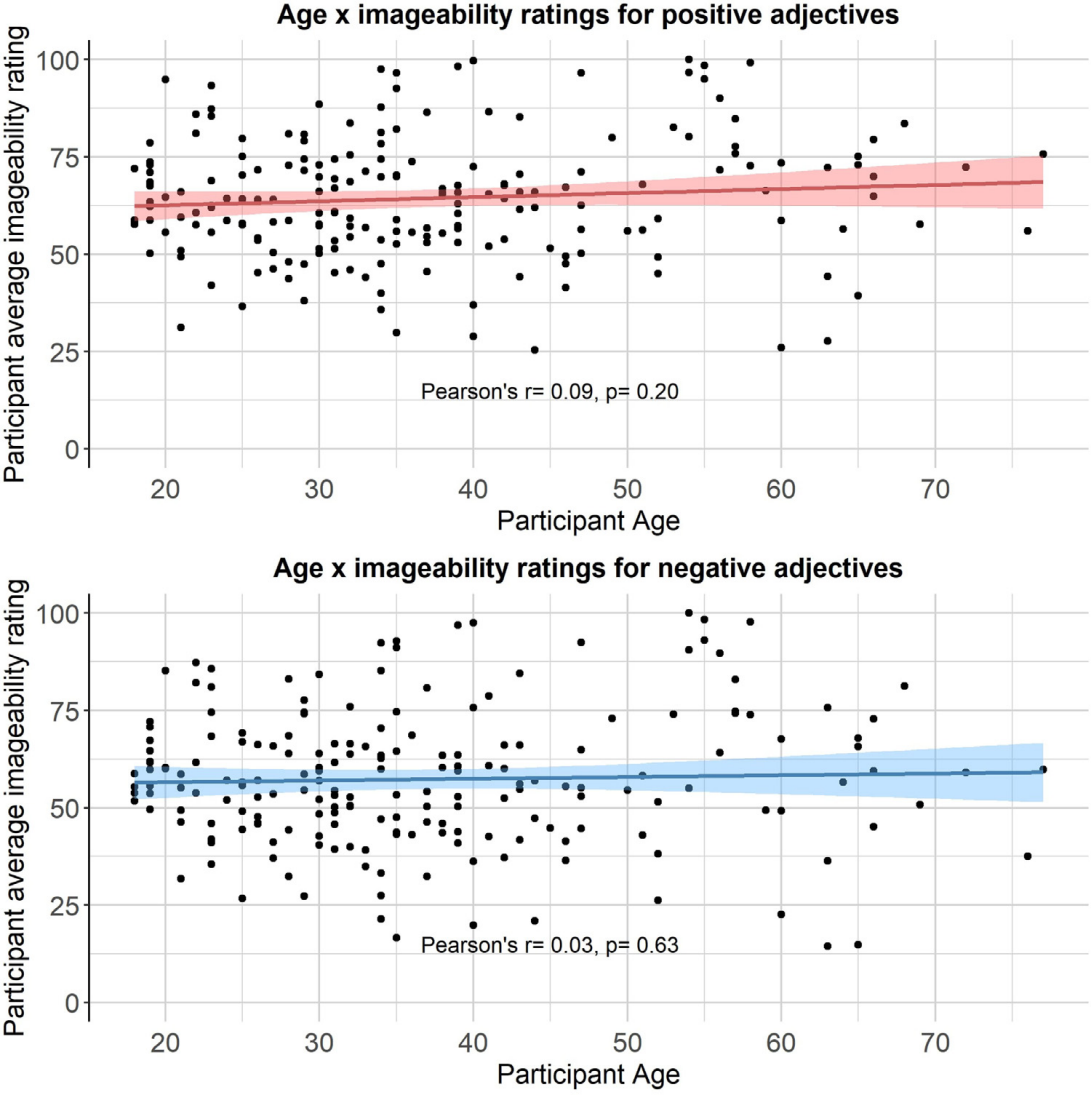
Correlations between participant imageability ratings for positive/negative adjectives and their age.

**Fig. 8:**
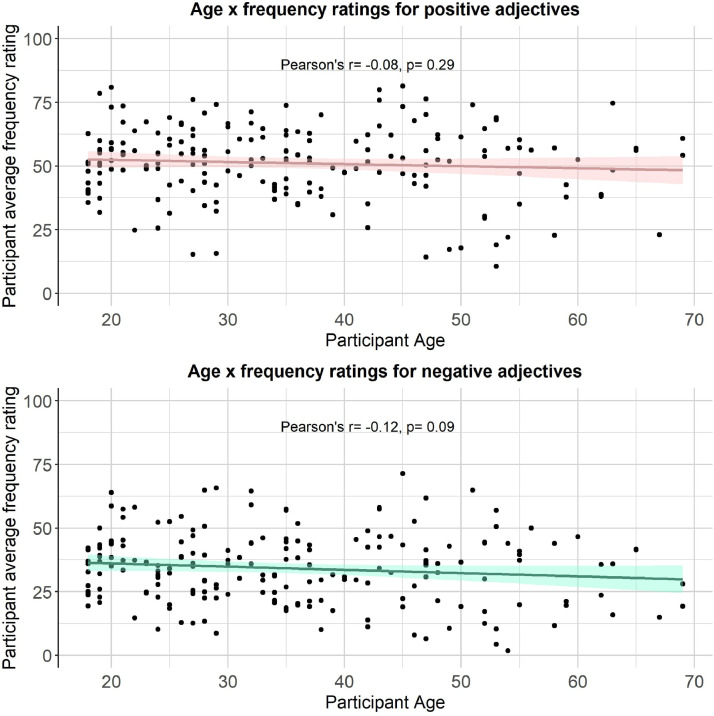
Correlations between participant subjective frequency ratings for positive/negative adjectives and their age.

We then computed a series of statistics (mean, standard deviation, standard error, number of ratings received, median, minimum rating, maximum rating, range, skew, kurtosis) for each type of rating for each of the 300 personality descriptors. The statistics for self-referential valence, imageability, subjective frequency and word length were merged into a final dataset (see Positive and negative personality descriptor words dataset). We pooled scores from all participants for the reported statistical analyses, based on exploratory analyses showing age, gender and depression/anxiety symptoms had little effect on participant ratings (see Fig. 2, Fig. 3, Fig. 4, Fig. 5, Fig. 6, Fig. 7, Fig. 8). However, if greater stratification is desired, specific population statistics can be re-calculated from the raw datasets.

**Fig. 9:**
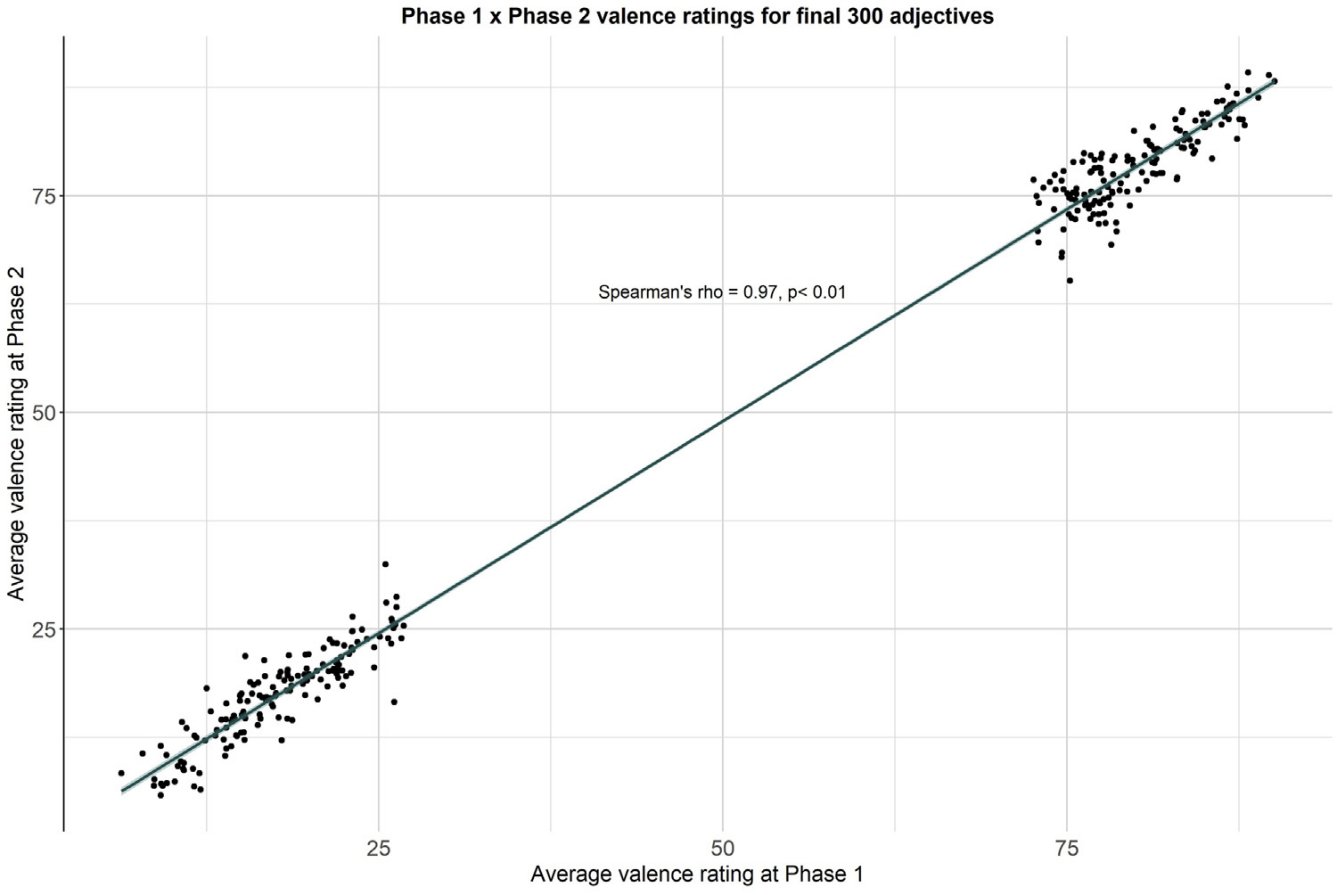
Correlation between mean self-referential valence ratings for final list of 300 adjectives, at Phase 1 vs Phase 2 of data collection.

We also explored the relationship between the initial self-referential valence ratings collected in Phase 1 (first Qualtrics survey) and those collected during Phase 2 (second Qualtrics survey) for our final list of 300 words. We found the mean ratings for each word to be highly correlated between the two surveys (Spearman's rho = 0.97, p < .01; see Fig. 9). Additionally, we conducted a mixed effects analysis of variance to statistically assess the effects of data collection phase on the self-referential valence ratings acquired for each personality descriptor (see Self-referential valence reliability dataset). After correction for multiple comparisons, the rating for only 1 of 300 words was found to differ significantly between Phase 1 and Phase 2 (“modern”).

No identifiable data was collected by the research team. Prolific academic collects identifiable data necessary for contacting participants, is GDPR compliant and does not store these data longer than is required. Only fully anonymised data is provided with this article – all pseudonymous variables have been removed by the research team prior to sharing.

## Ethics Statement

Collection and redistribution of these data was approved by the University of Oxford Central University Research Ethics Committee (CUREC), under reference number R71109/RE002. Informed consent was obtained from all participants prior to filling in the survey, and participant data are fully anonymised.

Table 1a, 1b

**Table 1a:** Participant demographics (all participants, including outliers).

Variable	Participant Sample 1, N=100^1^	Participant Sample 2, N=102^1^	Participant Sample 3, N=200^1^	Participant Sample 4, N=202^1^	p-value^2^
**Age**	33.64 (12.23)	37.25 (14.97)	36.84 (13.80)	35.36 (13.02)	0.3
**Gender**					0.8
Female	62 / 100 (62%)	56 / 102 (55%)	119 / 200 (60%)	120 / 202 (59%)	
Male	38 / 100 (38%)	44 / 102 (43%)	80 / 200 (40%)	80 / 202 (40%)	
Non-binary	0 / 100 (0%)	2 / 102 (2.0%)	1 / 200 (0.5%)	2 / 202 (1.0%)	
**Ethnicity**					0.7
Asian/British - Indian, Pakistani, Bangladeshi, other	8 / 100 (8.0%)	7 / 102 (6.9%)	9 / 200 (4.5%)	13 / 202 (6.4%)	
Black/Black British - Caribbean, African, other	5 / 100 (5.0%)	1 / 102 (1.0%)	10 / 200 (5.0%)	10 / 202 (5.0%)	
Chinese/Chinese British	1 / 100 (1.0%)	0 / 102 (0%)	4 / 200 (2.0%)	2 / 202 (1.0%)	
Middle Eastern/Middle Eastern British - Arab, Turkish, other	0 / 100 (0%)	0 / 102 (0%)	1 / 200 (0.5%)	1 / 202 (0.5%)	
Mixed race - other	3 / 100 (3.0%)	1 / 102 (1.0%)	8 / 200 (4.0%)	7 / 202 (3.5%)	
Mixed race - White and Black/Black British	0 / 100 (0%)	0 / 102 (0%)	5 / 200 (2.5%)	4 / 202 (2.0%)	
Other ethnic group	0 / 100 (0%)	1 / 102 (1.0%)	1 / 200 (0.5%)	0 / 202 (0%)	
Prefer not to say	1 / 100 (1.0%)	0 / 102 (0%)	1 / 200 (0.5%)	1 / 202 (0.5%)	
White - British, Irish, other	82 / 100 (82%)	92 / 102 (90%)	161 / 200 (80%)	164 / 202 (81%)	
**Highest education level**					0.5
A-levels or equivalent (at school till aged 18)	25 / 100 (25%)	22 / 102 (22%)	42 / 200 (21%)	58 / 202 (29%)	
Completed GCSE/CSE/O-levels or equivalent (at school till aged 16)	9 / 100 (9.0%)	16 / 102 (16%)	19 / 200 (9.5%)	13 / 202 (6.4%)	
Completed post-16 vocational course	6 / 100 (6.0%)	5 / 102 (4.9%)	13 / 200 (6.5%)	6 / 202 (3.0%)	
No qualifications	1 / 100 (1.0%)	1 / 102 (1.0%)	3 / 200 (1.5%)	1 / 202 (0.5%)	
Postgraduate degree	16 / 100 (16%)	20 / 102 (20%)	38 / 200 (19%)	34 / 202 (17%)	
Undergraduate degree or professional qualification	43 / 100 (43%)	38 / 102 (37%)	85 / 200 (42%)	90 / 202 (45%)	
**Income (in British Pounds)**					0.2
£16,000-£29,999 a year (£310-£569 a week)	22 / 100 (22%)	24 / 102 (24%)	58 / 200 (29%)	38 / 202 (19%)	
£30,000-£59,999 a year (£569-£1149 a week)	35 / 100 (35%)	32 / 102 (31%)	60 / 200 (30%)	72 / 202 (36%)	
£60,000-£89,999 a year (£1500-£1729 a week)	13 / 100 (13%)	20 / 102 (20%)	35 / 200 (18%)	37 / 202 (18%)	
£90,000-£119,999 a year (£1730-£2299 a week)	6 / 100 (6.0%)	7 / 102 (6.9%)	4 / 200 (2.0%)	8 / 202 (4.0%)	
Less than £16,000 a year (£310 a week)	11 / 100 (11%)	15 / 102 (15%)	25 / 200 (12%)	29 / 202 (14%)	
More than £120,000 a year (£2300 a week)	1 / 100 (1.0%)	0 / 102 (0%)	7 / 200 (3.5%)	4 / 202 (2.0%)	
Prefer not to say	12 / 100 (12%)	4 / 102 (3.9%)	11 / 200 (5.5%)	14 / 202 (6.9%)	
**English First Language**					<0.001
No	1 / 100 (1.0%)	0 / 102 (0%)	0 / 200 (0%)	0 / 202 (0%)	
No but bilingual	5 / 100 (5.0%)	3 / 102 (2.9%)	1 / 200 (0.5%)	1 / 202 (0.5%)	
No but fluent in English	6 / 100 (6.0%)	2 / 102 (2.0%)	0 / 200 (0%)	1 / 202 (0.5%)	
Yes	88 / 100 (88%)	97 / 102 (95%)	199 / 200 (100%)	200 / 202 (99%)	
**Dyslexia**					0.3
No	97 / 100 (97%)	94 / 102 (92%)	195 / 200 (98%)	196 / 202 (97%)	
Yes (diagnosed by a professional)	2 / 100 (2.0%)	5 / 102 (4.9%)	2 / 200 (1.0%)	4 / 202 (2.0%)	
Yes (self-diagnosed)	1 / 100 (1.0%)	3 / 102 (2.9%)	3 / 200 (1.5%)	2 / 202 (1.0%)	
**CESD total score**	16.52 (13.15)	15.83 (12.12)	17.31 (11.98)	19.41 (12.55)	0.043
**STAI-T total score**	43.42 (14.00)	43.27 (14.37)	44.77 (13.94)	47.21 (13.95)	0.038

1 Mean (SD); n / N (%)

2 Kruskal-Wallis rank sum test; Fisher's Exact Test for Count Data with simulated p-value (based on 2000 replicates)

**Table 1b:** Participant demographics (final sample, outliers removed).

Variable	Participant Sample 1, N=99^1^	Participant Sample 2, N=100^1^	Participant Sample 3, N=197^1^	Participant Sample 4, N=197^1^	p-value^2^
**Age**	33.73 (12.26)	37.38 (15.05)	37.09 (13.75)	35.34 (13.12)	0.2
**Gender**					0.8
Female	62 / 99 (63%)	55 / 100 (55%)	116 / 197 (59%)	116 / 197 (59%)	
Male	37 / 99 (37%)	43 / 100 (43%)	80 / 197 (41%)	79 / 197 (40%)	
Non-binary	0 / 99 (0%)	2 / 100 (2.0%)	1 / 197 (0.5%)	2 / 197 (1.0%)	
**Ethnicity**					0.7
Asian/British - Indian, Pakistani, Bangladeshi, other	8 / 99 (8.1%)	7 / 100 (7.0%)	8 / 197 (4.1%)	13 / 197 (6.6%)	
Black/Black British - Caribbean, African, other	4 / 99 (4.0%)	1 / 100 (1.0%)	9 / 197 (4.6%)	9 / 197 (4.6%)	
Chinese/Chinese British	1 / 99 (1.0%)	0 / 100 (0%)	4 / 197 (2.0%)	2 / 197 (1.0%)	
Middle Eastern/Middle Eastern British - Arab, Turkish, other	0 / 99 (0%)	0 / 100 (0%)	1 / 197 (0.5%)	1 / 197 (0.5%)	
Mixed race - other	3 / 99 (3.0%)	1 / 100 (1.0%)	8 / 197 (4.1%)	7 / 197 (3.6%)	
Mixed race - White and Black/Black British	0 / 99 (0%)	0 / 100 (0%)	5 / 197 (2.5%)	4 / 197 (2.0%)	
Other ethnic group	0 / 99 (0%)	1 / 100 (1.0%)	1 / 197 (0.5%)	0 / 197 (0%)	
Prefer not to say	1 / 99 (1.0%)	0 / 100 (0%)	1 / 197 (0.5%)	1 / 197 (0.5%)	
White - British, Irish, other	82 / 99 (83%)	90 / 100 (90%)	160 / 197 (81%)	160 / 197 (81%)	
**Highest education level**					0.6
A-levels or equivalent (at school till aged 18)	25 / 99 (25%)	21 / 100 (21%)	42 / 197 (21%)	56 / 197 (28%)	
Completed GCSE/CSE/O-levels or equivalent (at school till aged 16)	9 / 99 (9.1%)	15 / 100 (15%)	19 / 197 (9.6%)	13 / 197 (6.6%)	
Completed post-16 vocational course	6 / 99 (6.1%)	5 / 100 (5.0%)	13 / 197 (6.6%)	6 / 197 (3.0%)	
No qualifications	1 / 99 (1.0%)	1 / 100 (1.0%)	3 / 197 (1.5%)	1 / 197 (0.5%)	
Postgraduate degree	16 / 99 (16%)	20 / 100 (20%)	37 / 197 (19%)	32 / 197 (16%)	
Undergraduate degree or professional qualification	42 / 99 (42%)	38 / 100 (38%)	83 / 197 (42%)	89 / 197 (45%)	
**Income (in British Pounds)**					0.2
£16,000-£29,999 a year (£310-£569 a week)	22 / 99 (22%)	23 / 100 (23%)	57 / 197 (29%)	37 / 197 (19%)	
£30,000-£59,999 a year (£569-£1149 a week)	35 / 99 (35%)	32 / 100 (32%)	60 / 197 (30%)	70 / 197 (36%)	
£60,000-£89,999 a year (£1500-£1729 a week)	12 / 99 (12%)	20 / 100 (20%)	34 / 197 (17%)	36 / 197 (18%)	
£90,000-£119,999 a year (£1730-£2299 a week)	6 / 99 (6.1%)	7 / 100 (7.0%)	4 / 197 (2.0%)	8 / 197 (4.1%)	
Less than £16,000 a year (£310 a week)	11 / 99 (11%)	14 / 100 (14%)	24 / 197 (12%)	29 / 197 (15%)	
More than £120,000 a year (£2300 a week)	1 / 99 (1.0%)	0 / 100 (0%)	7 / 197 (3.6%)	4 / 197 (2.0%)	
Prefer not to say	12 / 99 (12%)	4 / 100 (4.0%)	11 / 197 (5.6%)	13 / 197 (6.6%)	
**English First Language**					<0.001
No	1 / 99 (1.0%)	0 / 100 (0%)	0 / 197 (0%)	0 / 197 (0%)	
No but bilingual	5 / 99 (5.1%)	3 / 100 (3.0%)	1 / 197 (0.5%)	1 / 197 (0.5%)	
No but fluent in English	6 / 99 (6.1%)	2 / 100 (2.0%)	0 / 197 (0%)	1 / 197 (0.5%)	
Yes	87 / 99 (88%)	95 / 100 (95%)	196 / 197 (99%)	195 / 197 (99%)	
**Dyslexia**					0.3
No	96 / 99 (97%)	92 / 100 (92%)	192 / 197 (97%)	191 / 197 (97%)	
Yes (diagnosed by a professional)	2 / 99 (2.0%)	5 / 100 (5.0%)	2 / 197 (1.0%)	4 / 197 (2.0%)	
Yes (self-diagnosed)	1 / 99 (1.0%)	3 / 100 (3.0%)	3 / 197 (1.5%)	2 / 197 (1.0%)	
**CESD total score**	16.52 (13.15)	15.43 (11.91)	17.27 (11.93)	19.43 (12.55)	0.028
**STAI-T total score**	43.42 (14.00)	42.82 (14.17)	44.68 (13.93)	47.38 (13.88)	0.018

1 Mean (SD); n / N (%)

2 Kruskal-Wallis rank sum test; Fisher's Exact Test for Count Data with simulated p-value (based on 2000 replicates)

## CRediT Author Statement

**Andreea Raslescu:** Conceptualisation, Investigation, Formal analysis, Data curation, Writing- Original draft. **Sophie Kreicker:** Methodology, Investigation, Formal analysis, Writing - Review & Editing. **Amy L Gillespie:** Conceptualisation, Validation, Writing - Review & Editing, Project administration. **William Berners-Lee:** Methodology, Investigation **Susannah E Murphy:** Conceptualisation, Writing- Review & Editing, Supervision. **Catherine Harmer:** Conceptualisation, Writing- Review & Editing, Supervision.

## Declaration of Competing Interest

The authors declare that they have no known competing financial interests or personal relationships which have or could be perceived to have influenced the work reported in this article.

## Data Availability

UK dataset of valence, imageability and frequency ratings of 300 adjectives for use in cognitive-emotional tasks (Original data) (Mendeley Data).
